# Characterisation of a New Fungal Immunomodulatory Protein from Tiger Milk mushroom, *Lignosus rhinocerotis*

**DOI:** 10.1038/srep30010

**Published:** 2016-07-27

**Authors:** V. Pushparajah, A. Fatima, C. H. Chong, T. Z. Gambule, C. J. Chan, S. T. Ng, C. S. Tan, S. Y. Fung, S. S. Lee, N. H. Tan, R. L. H. Lim

**Affiliations:** 1 Faculty of Applied Sciences, No. 1, Jalan Menara Gading, UCSI Heights, Cheras 56000 Kuala Lumpur, Malaysia; 2 Faculty of Pharmacy, Quest International University Perak (QIUP), No. 227, Plaza Teh Teng Seng (Level 2), Jalan Raja Permaisuri Bainun, 30250 Ipoh, Perak Darul Ridzuan, Malaysia; 3 Ligno Biotech Sdn. Bhd. No. 1, Jalan Perindustrian Balakong Jaya 2/2, Taman Perindustrian Balakong Jaya 2, 43300 Balakong Jaya, Selangor, Malaysia; 4 Horticulture Research Center, MARDI Headquarters, Serdang, P.O. Box 12301, 50774 Kuala Lumpur, Malaysia; 5 Department of Molecular Medicine, Faculty of Medicine, University of Malaya, 50603 Kuala Lumpur, Malaysia

## Abstract

*Lignosus rhinocerotis* (Tiger milk mushroom) is an important folk medicine for indigenous peoples in Southeast Asia. We previously reported its *de novo* assembled 34.3 Mb genome encoding a repertoire of proteins including a putative bioactive fungal immunomodulatory protein. Here we report the cDNA of this new member (FIP-Lrh) with a homology range of 54–64% to FIPs from other mushroom species, the closest is with FIP-glu (LZ-8) (64%) from *Ganoderma lucidum.* The FIP-Lrh of 112 amino acids (12.59 kDa) has a relatively hydrophobic N-terminal. Its predicted 3-dimensional model has identical folding patterns to FIP-fve and contains a partially conserved and more positively charged carbohydrates binding pocket. Docking predictions of FIP-Lrh on 14 glycans commonly found on cellular surfaces showed the best binding energy of −3.98 kcal/mol to N-acetylgalactosamine and N-acetylglucosamine. Overexpression of a 14.9 kDa soluble 6xHisFIP-Lrh was achieved in pET-28a(+)/BL21 and the purified recombinant protein was sequence verified by LC-MS/MS (QTOF) analysis. The ability to haemagglutinate both mouse and human blood at concentration ≥0.34 μM, further demonstrated its lectin nature. In addition, the cytotoxic effect of 6xHisFIP-Lrh on MCF-7, HeLa and A549 cancer cell lines was detected at IC_50_ of 0.34 μM, 0.58 μM and 0.60 μM, respectively.

In the same fungi family as Ling Zhi and Reishi mushroom, Tiger milk mushroom or scientifically known as *Lignosus rhinocerotis*, is one of the most popular medicinal mushrooms in Malaysia and specifically sought after by the *Semai* aborigine^1^. The mushroom’s sclerotium has been traditionally used for its medicinal properties to treat cough, fever, asthma, food poisoning and even prescribed for breast cancer^2^,^3^. Studies have reported very high amount of β-glucan and antioxidants in the sclerotium which may help to strengthen the body’s immune system and to suppress inflammation and metastasis of cancer cells^4^,^5^,^6^. Interestingly, these are similar to properties of a family of fungal immunomodulatory proteins (FIP), initially isolated from the fruit bodies or mycelia of higher Basidiomycetes.

Kino and colleagues^7^ were the first to report the isolation of FIP-glu or LZ-8 (GenBank: EU680479) from *Ganoderma lucidum* mycelia, subsequently other FIPs have been identified from different edible or medicinal mushrooms including FIP-fve from *Flammulina velutipes*^8^, FIP-gts from *Ganoderma tsugae*^9^, FIP-vvo from *Volvariella volvacea*^10^, FIP-gja from *Ganoderma japonicum*^11^, FIP-gmi from *Ganoderma microsporum*^12^, FIP-gsi from *Ganoderma sinense*^13^, FIP-gap from *Ganoderma applanatum*^14^ and FIP-tvc from *Trametes versicolor*^15^. Together, these formed the new family of FIP proteins that are small heat stable molecules of average molecular weight of 13 kDa (110–114 amino acids). The FIPs are acetylated at the N-terminus and are low in histidine, cysteine and methionine content but rich in asparagine and valine^16^. FIPs have similar structure and immunoregulatory activity to phytohaemagglutinin and immunoglobulins (Ig). FIP-fve and FIP-glu were reported to be identical to a heavy-chain variable region of immunoglobulin (Ig)^17^,^18^, essentially function in humoral immune response of mammals. It is capable of stimulating the proliferation of lymphocytes, monocytes and macrophages in mouse spleen, and enhancing production of cytokines by T helper type-1 (Th-1) cells, capable of inhibiting allergic reactions and anti-tumour activities^19^,^20^,^21^,^22^,^23^,^24^.

Computational studies can help to elucidate the structure and function of a new protein using simple homology modelling to predict the three dimensional (3-D) structure and docking techniques to determine its ligand binding sites with sufficient accuracy. The available 3-D structures of FIP-fve (1OSY.pdb), FIP-gmi (3KCW.pdb) from *G. microsporum* and FIP-glu (3F3H.pdb) show FIPs to occur constitutively as homo-dimers, arranged non-covalently in a dumb-bell-shaped similar to the variable region of Ig heavy chains^12^,^17^,^25^. The FIP-fve is folded into an Ig-like β-sandwich, each homodimer subunit consists of an N-terminal α-helix dimerization domain, followed by a fibronectin III (FNIII)-type fold. Dimerization is critical for the activity of the FIPs and occurs by swapping of the N-terminal helices and held strongly by hydrophobic interactions. Likewise, FIP-glu was found to resemble the structure of FIP-fve^17^,^25^ (Fig. 1). On the contrary, FIP-gmi, occurs as a tetramer instead of dimer and the arrangement of loops and conformation are significantly different from FIP-fve.

FIPs contain a carbohydrate binding module (CBM) in the β-sandwich at the C-terminal^26^. It is similar to the noncatalytic CBM family 34 (CBM-34), a β-sandwich folding family that acts as a granule starch-binding domain^26^,^27^. Therefore, FIP can bind to complex sugars such as dextrin, cyclodextrin and N-acetyl neuraminic acid on cell surface, which explains why majority of the reported FIPs are lectin in nature with different ability to agglutinate red blood cells (RBCs) from rat, mouse, sheep or human. Due to their small size, FIPs can be easily modified, with potential use in wide-ranging industrial applications^16^.

Recent genome sequencing of *L. rhinocerotis*^5^ revealed two putative FIPs (GME7566_g and GME10641_g) (FIP-Lrh), both exhibited 64% identity to FIP-glu. Here, we report the subsequent cloning and characterisation of the FIP-Lrh cDNA, including structure modelling for prediction of its putative carbohydrate binding module (CBM) and binding affinity to different cellular ligands. In addition, recombinant FIP-Lrh produced in *E. coli* was used to determine its haemagglutination ability and cytotoxicity on three cancer cell lines.

## Results and Discussion

### Isolation and cloning of FIP-Lrh cDNA

**Figure 2:**
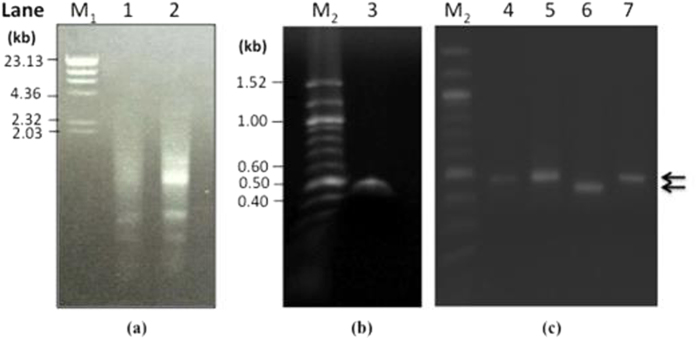
RT-PCR and cloning of FIP-Lrh cDNA. (**a**) Total RNA extracted from sclerotia of *L. rhinocerotis* as analysed on a 1% agarose gel electrophoresis. Lane M_1_ contained 1.5 μg of λ *Hin*dIII DNA marker (NEB, USA) whereas Lane 1 and 2 contained ~0.5 μg and 1.5 μg of total RNA. (**b**) An approximately 500 bp PCR product of FIP-Lrh cDNA was obtained through RT-PCR using FIPf and FIPr primers, as shown in Lane 3. M_2_ contained 1.25 μg of 100 bp DNA marker (NEB, USA). (**c**) Four pGEMT clones containing FIP-Lrh cDNA were subjected to PCR using FIPf and FIPr. A total of 5 μL PCR product obtained using clone pGEM_FIP_Lrh_1, pGEM_FIP_Lrh_2, pGEM_FIP_Lrh_3 and pGEM_FIP_Lrh_4 as template was loaded in Lane 4–7 respectively. Arrow indicates the presence of insert of approximately 400–500 bp in size.

**Figure 3:**
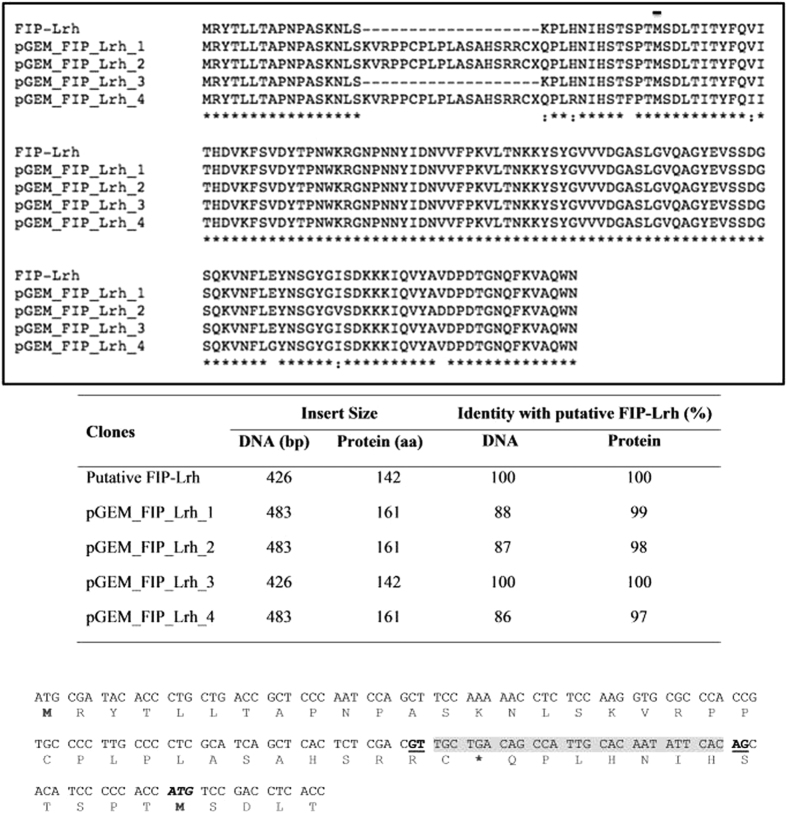
Multiple protein sequence alignment, protein and DNA sequence comparison of the four cloned FIP-Lrh cDNAs and the putative FIP-Lrh. The Met (M) initiation codon is as indicated (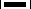). The position of the splice sites (donor, GT and acceptor, AG) in the intron is in bold and underlined and was predicted using the SplicePort tool (http://spliceport.cbcb.umd.edu). The predicted intron (27 bp) is shown in grey.

The extracted total RNA (yield of 0.153 μg/mg of sclerotia) (Fig. 2) when used in RT-PCR gave a PCR product of ~480 bp of the expected size which was subsequently cloned into pGEMT. Four recombinant clones were obtained, designated as pGEM_FIP_Lrh_1, pGEM_FIP_Lrh_2, pGEM_FIP_Lrh_3, and pGEM_FIP_Lrh_4. The pGEM_FIP_Lrh_3 gave a smaller sized PCR product (~450 bp) compared to the other three clones when subjected to PCR screening using the gene primer pairs (Fig. 2). The translated DNA sequence of inserts from the four clones showed that pGEM_FIP_Lrh_3 was 100% identical to the putative FIP-Lrh. The pGEM_FIP_Lrh_1, 2 and 4 contained an additional fragment of 18 amino acids (aa) followed by a stop codon which is probably part of an un-spliced intron of RNA processing due to the presence of a predicted acceptor splice site preceding the Met initiation codon (Fig. 3). Likewise, Zhou *et al.*^13^ reported the presence of a 61 bp intron at the 5′-flanking region preceding the Met initiation codon of FIP-gsi. Since the forward primer was designed to start at a methionine of the genomic putative FIP-Lrh ORF, untranslated sequences further upstream of the FIP gene was not available for further analysis.

**Figure 4:**
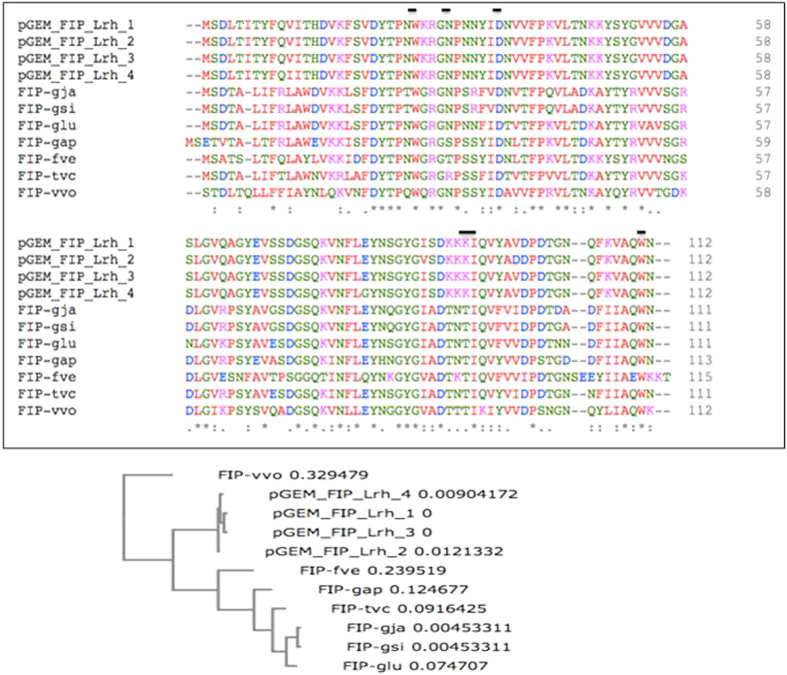
Multiple alignment of protein sequence and phylogeny of the four cloned FIP-Lrh cDNA with reported FIPs. The alignment is performed with the Clustal Omega (1.2.1) (EMBL-EBI) using the published FIP sequences of FIP-*gja* (*G. japonicum*) (GenBank: AAX98241.1), FIP-*gsi* (*Ganoderma sinense*), FIP-*glu* (*G. lucidum*) (UniProtKB/Swiss-Prot: P14945.2), FIP-gap (*G. applanatum*, GenBank: AEP68179.1), FIP-*fve* (*Flammulina velutipes*, GenBank: ADB24832.1), FIP-tvc (*Trametes versicolor*) and FIP-*vvo* (*Volvariella volvacea*) and gaps are introduced for optimal alignment and maximum similarity between all compared sequences. The identical amino acids among all the aligned sequences are indicated as ‘*’ whereas “:” conserved substitutions and empty space represents a non-conserved substitution. and the identical amino acids with FIP-*gsi* are shown in a gray background. The key residues in FIP-fve (W24, T28, D34, T90, I91 and W111) and the corresponding residues in FIP-Lrh (W25, D35, I92 and W112) present in the carbohydrate binding module (CBM) are shown as a solid line (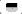). A Neighbour-joining phylogram (with real Branch length, distance corrected) was generated using the Omega Clustal program with FIPs from the representatives of genera Lignosus, Ganoderma, Flammulina, Volvariella and Trametes.

Reported FIP protein sequences from other mushroom species showed the putative Methionine (Met) initiation codon starts preceding a serine. Correspondingly, multiple alignment of protein sequences, starting from this putative Met, for the four cloned FIP-Lrh cDNA and the reported FIPs from seven mushroom species are as shown in Fig. 4. The pGEM_FIP_Lrh_2 exhibit one conserved amino acid substitution at position 87 (isoleucine to valine) and a non-conserved substitution at position 98 (valine to aspartic acid) while pGEM_FIP_Lrh_4 showed two substitutions, at position 11 (valine to isoleucine) conserved and at position 80 (glutamic acid to glycine) non-conserved, respectively. Since the pGEM_FIP_Lrh_1 and pGEM_FIP_Lrh_3 cDNA is 100% identical to the putative FIP-Lrh, it was used for subsequent analysis. The 29 amino acids fragment preceding the Met initiation codon of pGEM_FIP_Lrh_3 did not match to any signal peptides indicating that it may possibly be the 5′ untranslated region of the cDNA.

**Figure 5:**
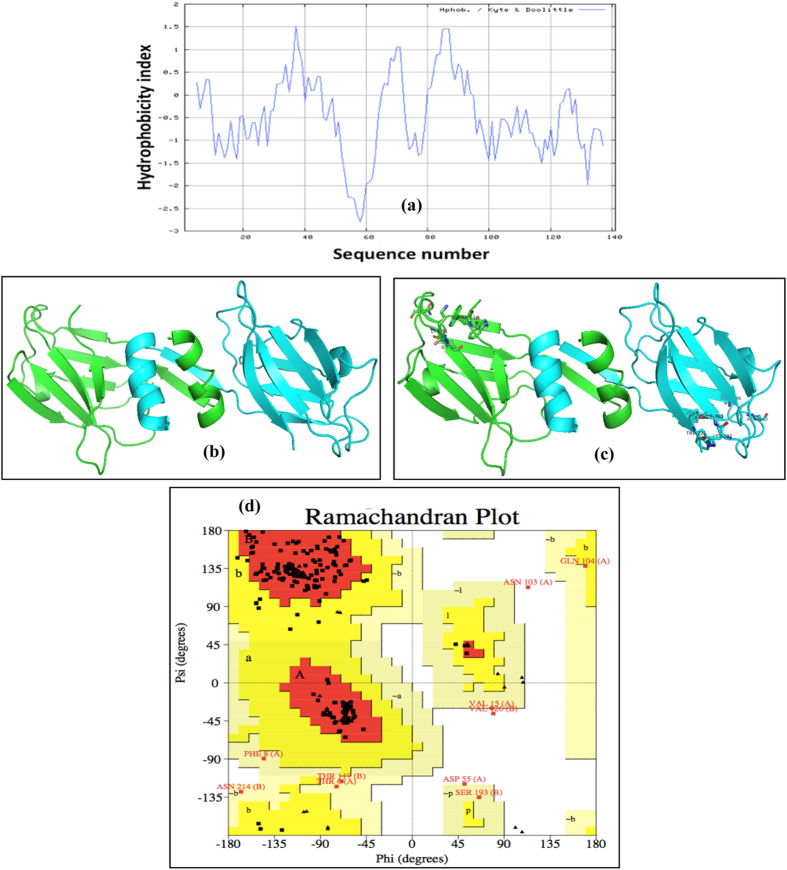
Hydrophobicity profile, predicted 3-D model of FIP-Lrh homodimer and Ramachandran plot. (**a**) The hydropathy plot of the deduced amino acid sequence of FIP-Lrh was plotted using the Kyte-Doolittle (http://web.expasy.org/protscale/). The 3-D structure of the homodimer showed α-helix at the N-terminal and the seven β-sheets (**a**,**b**).

Open reading frame (ORF) for the full-length FIP-Lrh cDNA encodes a protein of 112 aa (336 bp), with a calculated molecular mass of 12.59 kDa. Similar to mature FIPs from other mushrooms, FIP-Lrh contains a serine at the N-terminal that may be acetylated during post-translational modification. FIP-Lrh is predicted to be stable (instability index of −0.44, aliphatic index of 73.75) and contain mainly hydrophilic regions, except for the relatively hydrophobic middle region of the protein (Fig. 5a), unlike other FIPs with relatively hydrophobic N-terminals. Similar to other FIP proteins, it contains no Cys, Met (except for the initiation codon) or His residues, but it contains numerous Asp (n = 9), Asn (n = 10), Lys (n = 10), Ser (n = 9), Gly (n = 9), Tyr (n = 9) and Val (n = 15) residues. The FIP-Lrh cDNA exhibited 54–64% identity to reported FIPs, highest identity is to FIP-glu (LZ-8) (63.96%), followed by FIP-tvc (59.46%), FIP-gap (58.56%), FIP-gsi (58.56%), FIP-gja (57.66%), FIP-fve (54.05%) and FIP-vvo (53.57%). Correspondingly, a phylogeny analysis using the protein sequences revealed that the aligned FIP from *Lignosus* (FIP-Lrh cDNA) arise from a closer common ancestor with FIPs from *Flammulina, Ganoderma and Trametes* compared to that for *Volvariella* (Fig. 4). As previously reported, the FIP-gsi and FIP-gja were clustered into one subgroup, suggesting a close relationship^13^.

### Structure modelling and ligand docking

Since the functional form of the FIP-Lrh is a homodimer, the three dimensional structure of FIP-Lrh was predicted as a homodimer (Fig. 5b). It had 53.6% sequence identity and root mean square deviation score of 0.38 Å with its template FIP-fve (1OSY.pdb). The discrete optimised energy (DOPE) score of the predicted structure was −22852.3 kcal/mol which is an indication of the stability of the structure. The Ramachandran plot (Fig. 5d) of the predicted structure showed the bond length and angles were within limits with 87% of the amino acids in the core region. Four o-linked glycosylation were also predicted on each monomer. Ser1, Thr4, Thr6 and Ser58 of each monomer were predicted to be O-linked while none N-linked glycosylation sites were predicted due to lack of consensus Asn-Xxx-Ser/Thr sites^28^,^29^.

**Figure 6:**
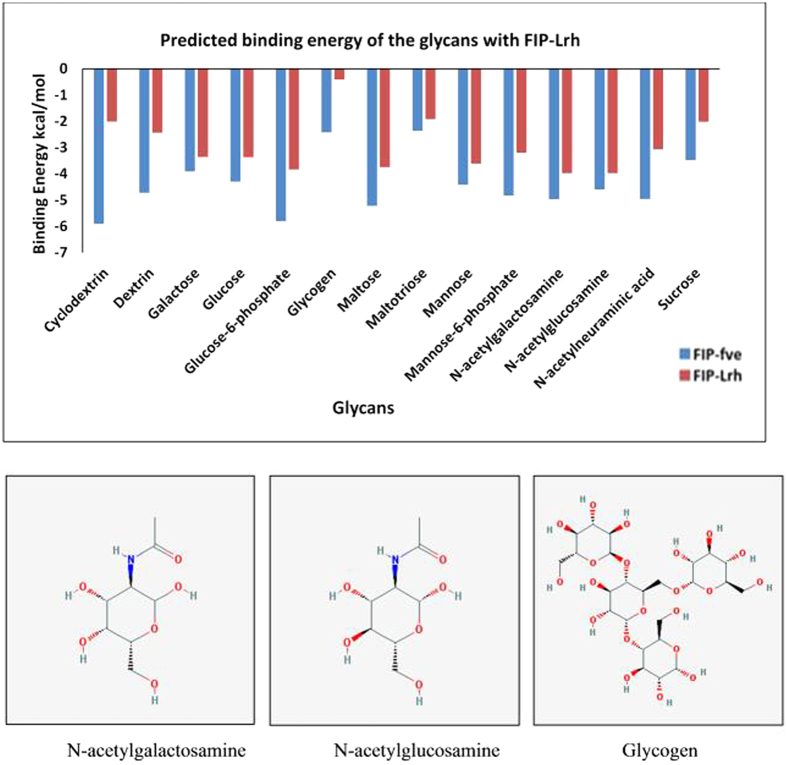
Comparison of the predicted binding energy of FIP-Lrh and FIP-fve to 14 different glycans. The structure of two glycans with the most stable binding, N-acetylgalactosamine and N-acetylglucosamine and the least binding glycan, Glycogen, are as shown.

**Figure 7:**
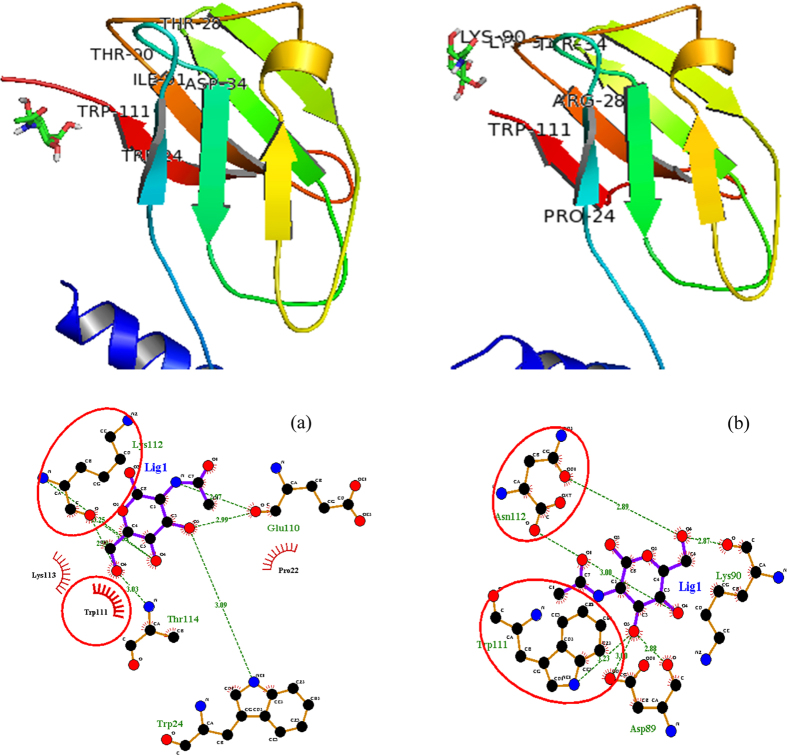
N-acetylgalactosamine docking into the active binding pocket of (**a**) FIP-fve and (**b**) FIP-Lrh and their corresponding LIGPLOT. The residues involved in binding of N-acetylgalactosamine are identified and are labelled for the 3-D structures of both proteins. Protein residues that are in equivalent 3D positions when the two structures are superposed are circled in red. H-bonds and their distances are indicated in green dashed lines. Residues closest to the ligand (indicated by stick and ball figures) whereas protein residues making hydrophobic interactions with ligand are indicated by spoked arcs.

The N-terminal of FIP-Lrh monomer forms a single α-helix joined to seven β sheets sandwiched at the C-terminal via a loop, similar to structures of FIPs reported earlier^17^. The β-sheet residues fold in immunoglobulin-like (Ig) domain indicating the functional similarity of FIP-Lrh to cell adhesion proteins. However it lacks Cys, Met and His residues usually found in Ig structures^26^. As reported previously by Liu *et al.*^26^, the β-sandwich of FIP-Lrh contains the CBM-34 domain; the identified six key residues of W24, T28, D34, T90, I91, and W111 in this domain of FIP-fve monomer bind to glycans on the membrane of human peripheral blood mononuclear cells (hPBMCs), promoting production of IFN-γ by T-cells. Four of these key residues were also present in FIP-Lrh. However their relative position was slightly different such as W25, D35, I92 and W110, whereas two residues, T28 and T90 of FIP-fve, were substituted by a hydrophilic and polar N29 and a positively charged K91 respectively, at comparable positions in FIP-Lrh (Fig. 5c, green coloured chain). Drawing on the experimental results of FIP-fve by Liu *et al.*^26^ subsequent docking analysis using glycans predicted that FIP-Lrh binds galactose, glucose, glucose-6-phosphate, maltose, mannose, N-acetylgalactosamine and N-acetylglucosamine with comparable binding energy as FIP-fve (Fig. 6). The CBM-34 binding pocket of FIP-fve comprised of hydrophobic and neutral amino acids and was experimentally demonstrated to bind strongly to N-acetylneuraminic acid, maltriose, cyclodextrin and dextrin, important for eliciting immune responses^26^. Comparatively, FIP-Lrh showed overall lower binding energies towards tested glycans especially for cyclodextrin, dextrin and N-acetylneuraminic acid. Among the 14 glycans, FIP-Lrh showed the best binding to N-acetylglucosamine and N-acetylgalactosamine with binding energy of −3.98 kcal/mol. Presence of a more positively charged CBM-34 binding pocket in FIP-Lrh as predicted by *in silico* analysis explained for the decreased interaction with cyclodextrin, dextrin and maltriose, while the negatively charged N-acetylgalactosamine and N-acetylglucosamine can bind strongly. This is in accordance with the results of Liu *et al.* who experimentally proved the substitution of the residues in the CBM-34 domain led to decreased immunomodulatory and haemagglutination activity, especially T28 resulted in little or complete abolition of IFN-γ in hPBMCs and haemagglutination activity while substitution of T90 resulted in reduced activity as compared to wild type. In FIP-Lrh these two crucial residues were replaced with arginine (N29) and lysine (K90) resulting in overall low binding energy prediction (Fig. 6)^26^. The LIGPLOT figure for comparison of protein-ligand complexes of FIP-fve and FIP-Lrh and N-acetylgalactosamine are as shown in Fig. 7.

### Expression and purification of 6xHisFIP-Lrh

**Figure 8:**
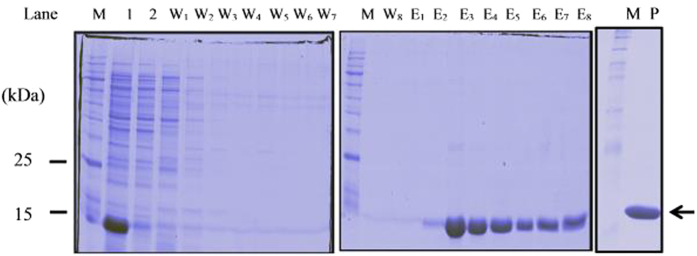
Ni-NTA purification of 6xHisFIP-Lrh protein. A total of 24 μL sample were loaded into each well, as follows: 1: cell lysate containing the soluble 6xHisFIP-Lrh protein; 2: contained the flow through collected after the Ni-NTA resin binding; W_1–2_, W_3–4_, W_5–6_, W_7–8_: contained flow through of washes in wash buffer with 20 mM, 30 mM, 40 mM and 50 mM Imidazole (pH8.0) respectively; the E_2–8_: eluents containing the 14.9 kDa recombinant 6xHisFIP-Lrh (arrow) eluted at 250 mM Imidazole. (P) the purified 6xHisFIP-Lrh (arrow). M: Broad range protein marker (p7710s, NEB, UK).

Further functional studies of FIP-Lrh can be facilitated by the availability of an active recombinant FIP-Lrh, since direct purification of this protein from the mushroom is costly, time-consuming and low in yield. The FIP-Lrh cDNA was cloned in-framed into pET-28a(+) and preliminary time-point total lysate profile of p2/BL21 expression culture is as shown in Fig. 8. Overexpression of a soluble 6xHisFIP-Lrh protein of approximately 14.9 kDa (including the 22 aa of the vector sequence and 6xHis tag) was observed at 1 hr after 1 mM IPTG induction, which was absent in the uninduced sample (t = 0). The amount of protein obtained increased as indicated by an increase in the intensity of the recombinant protein band with the duration of expression from 1 to 4 hr after induction. Purification of the 6xHisFIP-Lrh was performed under native condition where protein binding was optimal at pH 8.0, as minimal protein was found in the flow through from the resin (Fig. 8). The presence of imidazole (20–50 mM) in the binding buffer and wash buffer prevented binding of unspecific *E. coli* host proteins, therefore increasing the efficiency of the purification. Yield of the purified 6xHisFIP-Lrh was 9 mg/50 mL of expression culture and was used for subsequent haemagglutination and anti-proliferative assays.

### Verification of the identity of 6xHisFIP-Lrh via LC-MS/MS (QTOF)

The results of shotgun proteomics using LC-MS/MS (QTOF) showed that 14 peptides matched with the amino acid sequence of annotated immunomodulatory protein (accession number 10641) from *L. rhinocerotis* genome database with mean peptide spectral intensity of 3.21E+06 (Table 1). Peptide fragments from tryptic digested 6xHisFIP-Lrh covered 71.4% of amino acid sequence of the recombinant protein, or 85.6% of the annotated FIP protein (FIP-Lrh), thus establishing the identity of the recombinant protein as FIP.

### Haemagglutination and cytotoxicity of 6xHisFIP-Lrh

**Figure 9:**
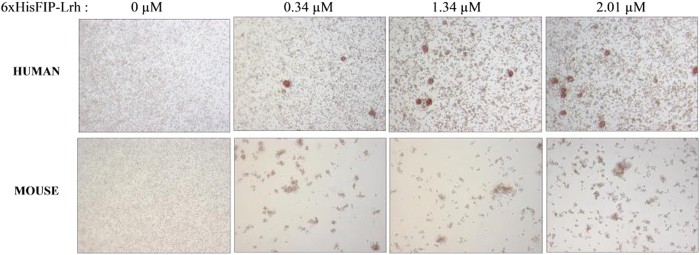
Haemagglutination assay of 6xHisFIP-Lrh. Different concentration of 6xHisFIP-Lrh (0–30 μg/mL, 0–2.01 μM) were incubated with 2% human or mouse RBCs and monitored under inverted microscope after 2 hr of incubation. The 6xHisFIP-Lrh was able to haemagglutinate both human and mouse whole blood at concentration of ≥0.34 μM (5 μ/mL), with greater degree of cells agglutination observed in mouse compared to human.

Results demonstrated that the 6xHisFIP-Lrh can agglutinate both human and mouse RBCs at concentration of ≥0.34 μM (5 μg/mL); greater degree of agglutination in mouse cells was observed compared to human cells (Fig. 9). Thus, similar to other FIPs, the FIP-Lrh is also part of a family of fungal lectins^30^ with a functional CBM binding pocket confirming its glycans binding ability predicted by *in silico* analysis.

**Figure 10:**
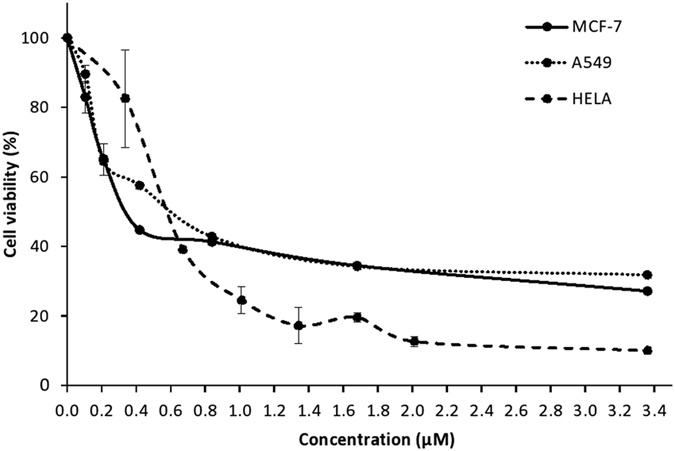
Cytotoxicity of 6xHisFIP-Lrh on human breast, cervical and lung cancer cell lines. Cell viability curve of 6xHisFIP-Lrh (0–3.36 μM) treated MCF-7 (ATCC HTB-22), A549 (ATCC CCL-185) and HeLa (ATCC CCL-2) cancer cell lines was obtained from triplicate assays. The IC_50_ of MCF-7, HeLa and A549 cells was 0.34 μM, 0.58 μM and 0.60 μM of recombinant protein, respectively.

In addition, preliminary investigation also showed that 6xHisFIP-Lrh exhibited a dose dependent cytotoxic effect on HeLa and A549 cells with an IC_50_ of approximately 0.58 μM (8.64 μg/mL) and 0.60 μM (8.94 μg/mL), respectively, whereas a lower IC_50_ of 0.34 μM (5.07 μg/mL) was observed for MCF-7 cells (Fig. 10). The positive control with DMSO, likewise showed dosage dependent cell killing (IC_50_ = 2.5–3.2%). This is in accordance to the cytotoxic effect on specific cancer cells lines reported for other recombinant FIPs. FIP from *Nectria haematococca* (FIP-nha) showed strong dose dependent antitumor effect on human gastric cancer MGC823 and liver cancer HepG2 cell lines with IC_50_ values of 15.54 and 12.24 μg/mL, respectively^31^. The rLZ-8 inhibited cancer cell proliferation by increasing G1 arrest^32^,^33^,^34^, rFIP-gts suppressed human alveolar A549 cells proliferation via the p53 activation pathway^35^ whereas FIP-gmi inhibited the metastatic ability of A549 cells^36^. Suppression of tumor cell growth by these FIPs is associated with an increase in poly functional T cells that secrete multiple effector cytokines, such as IL-2, IFN-γ and IL-17^37^,^38^,^39^.

The activity of a protein may be influence by proper protein folding, and post-transcriptional carbohydrate modification. Native FIPs (except LZ-8) were reported to lack carbohydrate modifications^8^. Recombinant FIPs, for example the rFIP-gts produced in insect cells^40^, rLZ-8 and rLZ-9 in yeast cells^41^, FIP-tvc^15^ and FIP-fve (with a His-tag)^26^ in *E. coli* lacks glycosylation albeit significant biological activity, indicating that carbohydrate modification of FIP is not necessary for biological activity. In accordance, the 6xHisFIP-Lrh expressed in *E. coli* showed substantial haemagglutination and cytotoxic properties. The underlying mechanism for its anti-tumour effects may be associated with immune cells activation through binding of CBM on their cellular glycans which need further experimental verification.

## Conclusion

A new FIP-Lrh cDNA was isolated from *L. rhinocerotis*, with closest protein sequence identity to *G. lucidum* (64.55%) and very similar predicted 3-D structure to FIP-fve. Like other FIPs, it is a sugar binding protein, and the more positively charged putative CBM pocket of FIP-Lrh predicted a stronger interaction with N-acetylgalactosamine and N-acetylglucosamine. A functional recombinant 6xHisFIP-Lrh was successfully produced in *E. coli* cells. This can facilitate future study to verify and examine the role of key residues in the binding of ligand-like glycoproteins on the surface of immune cells, to confer immunomodulatory properties for preventive and therapeutic potentials, such as anti-anaphylaxis and anti-tumor effect. The FIP-Lrh potential can therefore be explored for the development and utilization of medicinal proteins.

## Method

### Isolation and cloning of the FIP-Lrh cDNA

A total of 100 mg fresh sclerotia culture of *L. rhinocerotis* obtained from Ligno Biotech Sdn. Bhd. was homogenised in liquid nitrogen and total RNA was extracted using the NucleoSpin^®^ RNA Plant extraction kit (Macherey-Nagel, USA). SuperScript^®^ III First-Strand Synthesis System for RT-PCR (Invitrogen, USA) was used for cDNA synthesis according to manufacturer’s instruction. Briefly, 2 μg of extracted total RNA, 1 μL of dNTPs and 1 μL of 50 μM Oligo(dT)_20_ in a total 10 μL reaction volume was incubated at 65 °C for 5 min and placed on ice for 1 min. Then, 10 μL cDNA synthesis mix (2 μL 10x RT buffer, 4 μL 25 mM MgCl_2_, 2 μL 0.1 M DTT, 1 μL RNaseOUT^™^ and 1 μL SuperScript^®^ III RT) was added and RT reaction was performed at 50 °C for 50 min, terminated at 85 °C for 5 min and RNA was removed by incubating with 1 μL of 2 U/μL RNase H at 37 °C for 20 min. Specific PCR primers of forward FIP-f (5′-ATGCGATACACCCTGCTGACCG-3′; T_m_ = 61.4 °C) and reverse FIP-r (5′-CTAGTTCCACTGAGCG ACCTTGAAC-3′; Tm = 61.2 °C) were designed based on the putative coding genomic sequence of FIP-Lrh (Yap *et al.*^5^) and used for amplification of the FIP-Lrh cDNA in a total 25 μL reaction volume containing FIP-f and FIP-r (10 pmol each), 2 μL cDNA template, 1 U of *Taq* DNA polymerase, 0.2 mM dNTPs and 2.5 μL of 10x *Taq* buffer. PCR was performed in the Mastercycler^®^ Thermal Cycler (Eppendorf) with initial denaturation at 95 °C for 5 min; 25 cycles of denaturation at 95 °C for 30 sec, annealing at 60 °C for 30 sec, extension at 72 °C for 1 min; and a final extension at 72 °C for 5 min. The PCR product was gel purified using the Wizard^®^ SV Gel and PCR Clean-Up System (Promega, USA) and cloned using the pGEM^®^-T Easy Vector System (Promega, USA). White colonies were picked for plasmid DNA extraction using the Wizard^®^ Plus SV Minipreps DNA Purification System (Promega) and recombinant plasmids from four positive transformants were sent for sequencing of both DNA strands at MyTAGC Bioscience Sdn. Bhd. One of the positive transformants, designated as FIP-pGEM-T_3, was used as a template for PCR cloning into the NheI and HindIII site of pET28a(+) expression vector. Ligation mixture was transformed into BL21 strain *E. coli* cells and selected on Luria Bertani (LB) agar supplemented with 50 μg/mL kanamycin. Recombinant clones were then verified by PCR screening and DNA sequencing as previously described.

### *In silico* analysis of FIP-Lrh

DNA sequence analyses were performed using the NCBI’s Basic Local Alignment Search Tool (BLAST) on the NCBI (www.ncbi.nlm.nih.gov) website. The amino acid sequence of FIP-Lrh was deduced with DNA tools 5.0. The analysis and multiple alignment of the deduced amino acid sequence with published sequences of FIPs were performed using the blastp (standard protein-protein BLAST) and CLUSTAL O (1.2.1) on the EMBL-EBI (http://www.ebi.ac.uk/Tools/msa/clustalo/). The three-dimensional structure predictions of FIP-Lrh, its carbohydrate binding site(s) and important residues, homology modelling were done using the MODELLER 9 ver. 13^42^ while the docking analysis was using the Autodock 4.2^43^ and Autodock Vina^44^. Briefly, the structure of the FIP-fve template 1OSY.pdb was downloaded from Protein Data Bank^45^ and its quality of checked using PROCHECK^46^. Using FIP-fve as control template docking was performed using structures of 14 carbohydrate ligands (cyclodextrin, dextrin, galactose, glucose, glucose-6-phosphate, sucrose, glycogen, mannose, mannose-6-phosphate, maltose, maltotriose, N-acetylgalactosamine, N-acetylglucosamine and N-acetylneuraminic acid) that were downloaded from the PubChem Database. The respective 2-D plots of interactions were obtained by using LIGPLOT^47^. The glycosylation sites were predicted by using NetOGlyc 4.0 server (http://www.cbs.dtu.dk/services/NetOGlyc/)^28^ and GlycoEP (http://www.imtech.res.in/raghava/glycoep/)^29^. The FIP-monomer sequence was submitted to the server in FASTA format and the results obtained were subsequently analysed.

### Expression and purification of 6xHisFIP-Lrh

For expression study, 1 mL overnight culture of BL21 cells containing FIP-Lrh in pET-28a(+) expression vector (FIP-Lrh_pET/BL21) was used to inoculate 20 mL of LB broth (50 μg/ml Kanamycin) and allowed to grow at 37 °C with shaking at 150 rpm. At optical density (OD_600nm_) of 0.6, 1 mL of uninduced (t = 0 hr) culture sample was obtained and expression was induced by addition of 1 mM IPTG. Induced culture samples were obtained at hourly time points (t = 1 to 4 hr), the cells were pelleted by centrifugation at 12100 × g for 2 min and used for total lysate and solubility test as previously described^48^. The supernatant and cell pellet were collected as soluble and insoluble fractions for analysis on a 12% SDS-polyacrylamide gel electrophoresis (PAGE). Purification of the 6xHisFIP-Lrh recombinant protein was performed according to the manufacturer’s instructions with a few modifications (Invitrogen 2006). Cell pellet from 50 ml FIP-Lrh_pET/BL21 culture (at 2 hr after induction) was resuspended in 10 mL of native buffer (250 mM NaH_2_PO_4_, 0.5 M NaCl and 0.1 mM PMSF, pH8) and sonicated for 10 min of 10 s ON with 30 s OFF, at 160 W 20 kHz. The lysate was centrifuged at 4,500 rpm, 4 °C for 15 min and the supernatant containing the recombinant protein was purified using the Ni-NTA affinity chromatography. Briefly, the supernatant was incubated with 1 mL pre-equilibrated Ni-NTA resin for 1 hr and loaded on the Poly-Prep chromatography column (Biorad USA). It was washed with 4 mL each of wash buffer (50 mM NaH_2_PO_4_ and 0.5 M NaCl, pH8.0) containing 20 mM, 30 mM, 40 mM and 50 mM imidazole respectively. The purified 6xHisFIP-Lrh was eluted in 4 mL elution buffer (50 mM NaH_2_PO_4_, 0.5 M NaCl, 250 mM imidazole, pH8.0) and dialysed in three changes of 500 mL PBS (137 mM NaCl, 2.7 mM KCl, 10 mM Na_2_HPO_4_, 1.76 mM KH_2_PO_4_, pH 7.4) for 3 hr intervals each and another 3 hr on the following day. The purified protein was quantified using Bradford assay^49^ and analysed on a 12% SDS-PAGE.

### Sequence verification of 6xHisFIP-Lrh via LC-MS/MS (QTOF)

SDS-PAGE band for identification was excised into gel plugs of 1 mm × 1 mm, destained with 50% acetonitrile in 50 mM ammonium bicarbonate at 37 °C, reduced with dithiothreitol (10 mM) in 100 mM ammonium bicarbonate at 60 °C for 30 min and alkylated with 55 mM iodoacetamide in 100 mM ammonium bicarbonate for 20 min in the dark at 37 °C. In-gel tryptic digestion of protein from SDS-PAGE was performed using Pierce™ Trypsin Protease, MS grade (Thermo Scientific, Massachusetts, USA) according to manufacturer’s instructions. Digested proteins were desalted with ZipTip® pipette tip (Merck Millipore) before analysis with an Agilent 1260 HPLC-Chip/MS Interface coupled with Agilent 6550 Accurate-Mass Q-TOF LC/MS.

Desalted protein sample was reconstituted with 7 μl of solution A (0.1% formic acid in water). Sample injection volume was 2 μl. After sample pre-concentration, analytical separation was accomplished over a 75 μm × 150 mm analytical column at 0.4 μl/min in an initial gradient of 5% solvent B, 50% solvent B at 11 min, 70% solvent B at 15 min, 70% solvent B at 18 min and 5% solvent B at 19 min. Solvent A consists of 0.1% formic acid in water while solvent B contains 100% acetonitrile in solvent A. Total run time was 25 min including post-run of 6 min. For subsequent MS (rate: 8 spectra/s, time: 50 ms/spectrum) and MS/MS (rate: 4 spectra/s, time: 125 ms/spectrum) analyses, spectra were acquired in a MSMS mode with scan range from 200 to 3000 m/z and 50 to 3200 m/z, respectively. Capillary and fragmentor voltage were 1800 V and 175 V, respectively with drying gas flow rate of 5.0 l/min at 290 °C. Generated raw data were searched against *L. rhinocerotis* genome database using Agilent Spectrum Mill MS Proteomics Workbench software packages. The following parameters and filters were implemented for protein and peptide identification: MH+ scan range from 100 to 3200 Da, carbamidomethylation of cysteines was set as a fixed modification, protein score >20, fast discovery rate <1%.

### Haemagglutination assay

In compliance with ethics standards, the methods used to obtain human and animal blood were performed in accordance with the guidelines and experimental protocols approved by the ethics committee for the Faculty of Applied Sciences, UCSI University. Informed consent was obtained from human blood donor and assay was performed according to Hsu *et al.*^50^ with a few modification. Briefly, a total of 100 μL whole blood was collected using a heparinized capillary tube from Balb/c mouse via retro-orbital route while human blood was collected via finger pricking. The blood samples were washed three times with equal volume of PBS and collected by centrifugation at 200 g for 10 min. A total 100 μL of 2% human or mouse blood in PBS was respectively co-incubated with 6xHisFIP-Lrh (0 to 2.01 μM) in a 96-well plate. The plate was shaken for 30 sec and incubated in a CO_2_ incubator for 2 hr at 37 °C and degree of haemagglutination was observed under an inverted microscope.

### Cytotoxicity assay on cancer cell lines

Human cervical cancer HeLa cell line (ATCC CCL-2) was cultured and maintained in Dulbecco’s Minimum Essential Medium (DMEM, Gibco) supplemented with 5% Fetal Bovine Serum (FBS). Human breast cancer MCF-7 cell line (ATCC HTB-22) and human lung cancer A549 cell line (ATCC CCL-185) were cultured and maintained in Roswell Park Memorial Institute 1640 medium (RPMI-1640, Biowest) supplemented with 10% FBS. All media were supplemented with penicillin (100 U/mL) and streptomycin (100 μg/mL) in cytotoxicity study. Cells were cultured at 37 °C in a 5% CO_2_ incubator.

Cells at 70% confluent were harvested by trypsinization and used for cytotoxicity assay as previously described^51^. Briefly, cells seeded in triplicate wells at 1 × 10^4^ cells/200 μl/well were added with 50 μl of 6xHisFIP-Lrh ranging from 0–3.36 μM. The positive control was DMSO (0% to 20%) and negative control was untreated cells. Cells were incubated for 24 hr overnight after which 50 μl of MTT solution (5 mg/mL) was added into each well and incubated further for 4 hr. The supernatant was then discarded, 200 μl of DMSO was added and optical density (OD) at 570 nm was measured. IC_50_ of 6xHisFIP-Lrh against respective cell lines was calculated from triplicate assays.

## Additional Information

**How to cite this article**: Pushparajah, V. *et al.* Characterisation of a New Fungal Immunomodulatory Protein from Tiger Milk mushroom, *Lignosus rhinocerotis. Sci. Rep.* **6**, 30010; doi: 10.1038/srep30010 (2016).

## Figures and Tables

**Figure 1:**
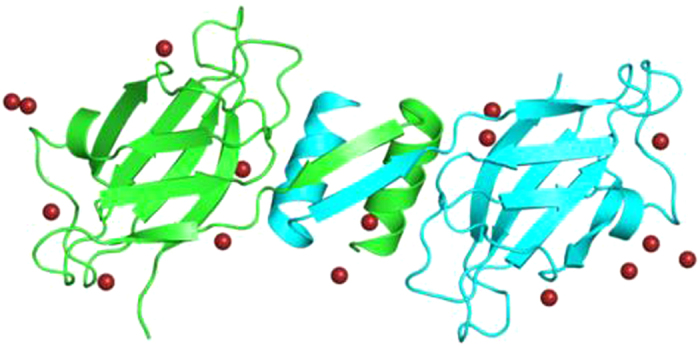
The structure of FIP-fve dimer (1OSY.pdb).

**Table 1:** LC-MS/MS report of tryptic digested 6xHisFIP-Lrh.

Accession number	Annotated protein name	Species	Molecular mass (Da)/Protein pI	Matched peptide fragments with annotated immunomodulatory protein (accession number 10641) from *L. rhinocerotis* genome database
10641	Immunomodulatory protein 8	*Ganoderma lucidum*	15759.4/9.01	IQVYAVDPDTGNQFK
				KKIQVYAVDPDTGNQFK
				GNPNNYIDNVVFPK
				KIQVYAVDPDTGNQFK
				FSVDYTPNWKR
				RGNPNNYIDNVVFPK
				VNFLEYNSGYGISDKKK
				VNFLEYNSGYGISDKK
				VNFLEYNSGYGISDK
				KYSYGVVVDGASLGVQAGYEVSSDGSQK
				YSYGVVVDGASLGVQAGYEVSSDGSQK
				IQVYAVDPDTGNQFKVAQWN
				GNPNNYIDNVVFPKVLTNK
				YSYGVVVDGASLGVQAGYEVSSDGSQKVNFLEYNSGYGISDK

There were 14 distinct matched peptides, with distinct summed MS/MS Search score of 269.12 and a mean peptide spectral intensity of 3.21E+06.
